# Assessment of neurological symptoms and associated factors in patients with Wilson’s disease in Southwest China

**DOI:** 10.1186/s13023-025-03874-2

**Published:** 2025-07-04

**Authors:** Lu Zhang, Jieru Peng, Yao Dong, Qiwen Zhang, Wencheng Long, Yueshan Wang, Zhong Li, Lu Long, Yaxin Li, Qiaoling Jin, Lin Deng, Lin Cai, Dailan Yang, Juan Liao, Chunxia Yang

**Affiliations:** 1 Department of Epidemiology and Biostatistics, West China School of Public Health and West China Fourth Hospital, Sichuan University, Chengdu, China; 2 Department of Toxicosis/Nephrology, West China School of Public Health and West China Fourth Hospital, Sichuan University, Chengdu, China; 3 Department of Gastroenterology, West China School of Public Health and West China Fourth Hospital, Sichuan University, Chengdu, China; 4 Non-Communicable Diseases Research Center, West China-PUMC C.C. Chen Institute of Health, Sichuan University, Chengdu, China

**Keywords:** Wilson’s disease, Neurological symptoms, Severity, Unified Wilson’s Disease Rating Scale (UWDRS)

## Abstract

**Background:**

Wilson’s disease is an inherited genetic disorder of hepatic copper metabolism characterized by hepatic, neurological, and psychiatric manifestations. Patients with neurological symptoms manifest remarkable variability regarding type and severity. This study aimed to characterize neurological signs and symptoms of patients with Wilson’s disease in Southwest China and identify factors associated with neurological symptoms in patients with Wilson’s disease.

**Methods:**

A total of 109 treated patients with Wilson’s disease were included in the study. Sociodemographic and clinical data were obtained through face-to-face interviews and medical record reviews. ATP7B mutations were identified through whole-genome resequencing. Neurological signs and symptoms were assessed using the neurological part of the Unified Wilson’s Disease Rating Scale (UWDRS Part I). Multiple linear regression analysis was performed to assess the association between patient characteristics and UWDRS Part I scores.

**Results:**

The most prevalent neurological symptoms of patients with Wilson’s disease were impaired rapid alternating movements of hands (76.2–81.0%), impaired finger tapping (75.0%), dysarthria (70.2%), salivation (66.7%), impaired handwriting (61.9%), impaired legs agility (60.7–61.9%), impaired gait (leg dystonia, 59.5%; ataxia, 58.3%), and dystonia of arms and hands (54.8–56.0%). Sex and age differences were observed in the neurological features of Wilson’s disease. Sociodemographic and clinical factors associated with the severity of neurological symptoms included occupation, family per capita monthly income, initial clinical subtype, adherence to low-copper diets, and mental health conditions, with an explanatory power of 42.1% (*F* = 10.474, *p* < 0.001). Genotype–phenotype analysis showed that patients carrying the p.P992L mutation had a significantly higher frequency of impaired finger tapping (*p* = 0.037).

**Conclusion:**

The main neurological symptoms in this study of treated patients with Wilson’s disease were lack of motor coordination, dystonia, dysarthria, and salivation. This study identified five factors associated with the severity of neurological symptoms and revealed a potential association between the p.P992L mutation and a specific neurological manifestation. These results may enhance the understanding of Wilson’s disease, guide future management of patients to alleviate neurological symptoms and improve prognosis.

**Supplementary Information:**

The online version contains supplementary material available at 10.1186/s13023-025-03874-2.

## Introduction

Wilson’s disease (WD) is a rare autosomal recessive genetic disorder of copper metabolism caused by pathogenic mutations in the ATP7B gene located on chromosome 13, which encodes a copper-transporting *P*-type ATPase (ATP7B). Defective ATP7B function leads to copper metabolism dysfunction and excessive copper accumulation, particularly in the liver and brain, resulting in the hepatic and neurological features of Wilson’s disease [1]. It has previously been estimated that there were about 30 cases of WD per 1 million population worldwide [2]. However, emerging data suggest that WD might be more common than previously estimated [3, 4].

Patients with WD manifest remarkable variability regarding type and severity, and may present as liver disease, neurological disorders, psychiatric disorders or a combination of these. Neuropsychiatric manifestations tend to occur 10 years later than hepatic manifestations [1, 5, 6]. In addition, a variety of clinical manifestations such as renal, cardiac, musculoskeletal, and endocrine conditions may occur [4].

40–50% of WD patients present with neurological symptoms, which usually appear around 20–30 years of age [1]. These symptoms may be the first clinical manifestation of WD appearing simultaneously with symptoms of liver disease, or appear some years later. The common neurological symptoms include dystonia, tremor, bradykinesia and rigidity, ataxia and others such as choreiform movements, seizures, insomnia and so on [1, 7, 8]. Patients may have more than one neurological manifestations and each abnormality varies in severity, which can fluctuate even daily during the course of disease [6, 7].

The Unified Wilson’s Disease Rating Scale (UWDRS) is recommended to objectively assess neurological status in WD patients, which is a descriptive neurological scoring system used to standardize neurological findings in WD patients [4, 6]. The UWDRS, initially developed in 2007 as a novel rating scale for WD by Członkowska A et al., aimed to accurately reflect the motor impairment of WD patients [9]. In 2008, Leinweber B et al. extended it with the addition of hepatic and psychiatric subscales. Currently, the full UWDRS consists of 3 subscales (55 items): neurological subscale (27 items), hepatic subscale (9 items), and psychiatric subscale (19 items) [10]. The neurological subscale consists of daily living as reported by the patient or their proxy and a detailed neurological examination, which is a valuable tool for the clinical assessment of neurological signs and symptoms in WD patients [11, 12].

Neurological manifestations of WD are intricate, and factors associated with the severity of neurological symptoms are understudied [7, 8, 13]. Furthermore, the UWDRS for assessment of neurological symptoms in patients with WD is underutilized in Chinese population [14–16]. In this study, we sought to characterize and evaluate the neurological signs and symptoms of WD patients using the neurological subscale of UWDRS and analyze factors associated with neurological symptoms.

## Methods

### Participants

This study was performed at the West China School of Public Health and West China Fourth Hospital of Sichuan University in Chengdu, Sichuan Province, China, which is the only hospital in Sichuan Province that can provide intravenous copper chelation therapy. At our center, in accordance with the Chinese guidelines for diagnosis and treatment of Wilson’s disease, hospitalized patients primarily receive intravenous dimercaptopropane sulfonate (DMPS) as a chelating agent. For those who cannot tolerate DMPS, calcium sodium edetate (EDTA) is used as an alternative. Additionally, patients receive liver-protective and symptomatic treatments [17, 18]. For maintenance therapy outside the hospital, patients take oral chelating agents, including D-penicillamine (DPA), dimercaptosuccinic acid (DMSA), and zinc (Zn). It is important to note that DMPS and DMSA are not currently included as first-line therapies in international guidelines. However, due to their low cost, good tolerability, and promising clinical efficacy observed in Chinese clinical practice, they are widely used in China [18, 19].The subjects of this study were inpatients who had a confirmed diagnosis of WD and came to our center for copper chelation therapy between August 2023 and April 2024.

In China, the diagnosis of WD is based on a combination of clinical features and laboratory parameters: age at onset (5–35 years old), symptoms (hepatic/neuropsychiatric symptoms), copper biochemical parameters (serum ceruloplasmin < 200 mg/L, urinary copper excretion ≥ 100 μg/24 h, or liver copper > 250 μg/g dry weight), urinary copper excretion > 1600 μg/24 h after the administration of 2 × 500 mg D-penicillamine, presence of Kayser-Fleischer ring by slit-lamp examination, positive family history, and mutation analysis. Patients with extrapyramidal/hepatic symptoms, positive K-F rings, serum ceruloplasmin < 200 mg/L, and urinary copper excretion ≥ 100 μg/24 h are diagnosed with WD and no further examination is required[17, 18]. Additionally, the Leipzig score was referenced [6].

The patients were excluded if they met any of the following criteria: (1) malignant tumors; (2) severe heart, pulmonary, or renal disease; (3) other hepatic diseases such as alcoholic, viral, autoimmune, drug-induced, or parasitic liver disease; (4) other neurological diseases such as Parkinson’s disease, primary tremor, or Huntington’s disease; (5) severe mental disorders; (6) important medical data missing or (7) unwillingness to participate in this study.

This study was performed with the approval of the Ethics Committee of West China School of Public Health and West China Fourth Hospital (Gwll2023126) and adhered to the Declaration of Helsinki. All the written informed consent was obtained from participants. We confirm that all methods were executed according to the relevant guidelines.

### Measurements

#### Sociodemographic and clinical characteristics

This study collected data through face-to-face interviews and medical record reviews. The questionnaire was developed by a team consisting of the study leaders, co-researchers, clinicians, and research assistants. Questions were developed from existing surveys, generally accepted scales, and group discussions (Supplementary Table 1). The following sociodemographic and clinical characteristics of the participants were used from the survey data and medical records: demographic characteristics (sex, age, residence, body mass index (BMI), educational level, marital status, occupation, per capita monthly income), lifestyle factors (physical exercise, sleep duration and quality, smoking and drinking status), disease-related information (family history, age at onset, initial clinical subtypes, duration from onset to diagnosis, history of misdiagnosis, disease duration, years of treatment), treatment and adherence (intravenous chelating agent, oral chelating agent, adherence to regular medication, regular review, and low-copper diets), psychosocial factors (social support, mental health conditions), liver function, and copper metabolism parameters.

Social support was measured using the Social Support Rating Scale (SSRS) [20]. A score of 45–66 on the scale indicates high social support, a score of 23–44 indicates medium social support and a score of 12–22 indicates low social support. Psychological distress was measured using the Kessler Psychological Distress Scale (K10) [21]. A score of 10–15 on the scale indicates good mental health, a score of 16–21 indicates moderate mental health, a score of 22–29 indicates poor mental health and a score of 30–50 indicates bad mental health [22].

Additionally, this study utilized Child-Turcotte-Pugh (CTP) score/class and the Albumin-Bilirubin (ALBI) score/grade to assess patients’ liver function. CTP score was calculated using prothrombin time, albumin, bilirubin, and clinical findings of ascites and encephalopathy [23], and it was categorized into 3 classes: class A (5–6), B (7–9), and C (10–15). ALBI score was calculated using the formula: ALBI = log10 (bilirubin in µmol/L) × 0.66 + albumin in g/L × (− 0.085) [24]. ALBI score was stratified into 3 grades: ALBI grade 1 (≤ − 2.60), ALBI grade 2 (> − 2.60 but ≤ − 1.39), and ALBI grade 3 (> − 1.39).

#### Neurological symptoms

All participants in this study had their symptoms comprehensively assessed using the UWDRS within the first 2 days of admission by researchers who were professionally trained by clinical neurologists [10]. The UWDRS was developed as a tool to comprehensively assess the clinical symptoms of WD patients, including neurological (Part I, 27 items), hepatic (Part II, 9 items), and psychiatric (Part III, 19 items) clinical signs of WD. Each item was assessed on a five-point scale ranging from 0 (asymptomatic) to 4 (most severe). This study used the UWDRS Part I score to assess the severity of neurological symptoms in patients with WD.

#### Mutation analysis of the ATP7B gene

Genomic DNA was extracted from peripheral blood samples of patients with WD. Whole-genome resequencing was performed by Biomarker Technologies Co., Ltd (Beijing, China), following standard protocols for DNA extraction, library construction, high-throughput sequencing, and data analysis. This study focused on mutations within the 21 exons of the ATP7B gene. Sequencing reads were aligned to the reference sequences (NCBI accession NM_000053.4/Ensembl accession ENST00000242839.10) to identify mutations. The pathogenicity of the mutations was assessed using InterVar (http://wintervar.wglab.org/) and the ClinVar database (https://www.ncbi.nlm.nih.gov/clinvar/). Pathogenic mutations in this study were defined as mutations classified as pathogenic or likely pathogenic by InterVar or ClinVar. For mutations without available classification information, pathogenicity was inferred based on the following criteria: (1) predicted to be damaging by SIFT (https://sift.bii.a-star.edu.sg/) and PolyPhen-2 (http://genetics.bwh.harvard.edu/pph2/index.shtml); and (2) identified as loss-of-function (LOF) mutations, defined as nonsense, frameshift, or splice-site mutations that are considered severely disruptive to ATP7B protein function [25].

### Statistical analysis

SPSS (version 26.0) and R (version 4.3.3) were used for data analysis. Results were presented as frequencies and proportions for categorical variables, median (interquartile range) for non-normally distributed continuous variables, and mean ± standard deviation (SD) for normally distributed continuous variables. Chi-square test or Fisher’s exact test (if the expected frequency was < 5) was used for group comparisons of categorical variables. Comparisons of total scores and item scores of UWDRS Part I were analyzed using the Mann–Whitney test in the case of 2 groups, while inter-group comparisons for more than two groups were analyzed using the Kruskal–Wallis test. Pairwise comparisons between the groups were performed using Dunn’s test with Bonferroni correction for *p*-value adjustment. The stepwise multiple linear regression and its determination coefficient (*R*^*2*^) were used to identify factors independently associated with the severity of neurological symptoms. Two-sided *p*-values less than 0.05 were considered statistically significant.

## Results

### Patient characteristics

#### Sociodemographic characteristics

Among the 109 patients with WD included in the analysis, 84 (77.1%) had neurological symptoms at enrollment, and 25 (22.9%) did not. The sociodemographic and clinical characteristics of these patients are summarized in Supplementary Table 2. Most patients were female (56.0%), 21–40 years of age (73.4%), rural residents (62.4%), in the BMI range of 18.5–24 kg/m^2^ (55.0%), and with an education level of senior high school and above (61.5%). Additionally, 45.9% of the patients were unmarried, 47.7% were unemployed, and 39.4% had a monthly per capita household income below CNY2500.

Regarding lifestyle habits, most patients reported that they exercised less than once per week (52.3%), slept 6–7 h per night (52.3%), and had good sleep quality (55.0%) over the past 6 months. Furthermore, the vast majority did not smoke (90.8%) or drink alcohol (87.2%).

#### Clinical characteristics

Among the patients, 25.7% had a positive family history of WD, with most affected relatives being siblings (89.3%). The median age at onset was 18 years (range 3–53 years), and 61.5% initially presented with the neurologic subtype. While the majority (68.8%) were diagnosed within a year of symptom onset, more than half (54.1%) had experienced a prior misdiagnosis. Among the 59 patients with a history of misdiagnosis, they were primarily misdiagnosed with conditions such as liver diseases (49.2%), mental disorders (20.3%), joint diseases (6.8%), or encephalopathy (6.8%). The median disease duration was 10 years (range 1–30 years), while the median duration of treatment was 9 years (range 0–28 years).

During hospitalization, the intravenous copper chelator used in most patients was DMPS (90.8%). For long-term oral chelation therapy, patients took DPA, DMSA, and/or Zn in various combinations. Specifically, 21.1% received a combination of DPA and Zn, while 26.6% were treated with DMSA and Zn. Additionally, 16.5% used DPA alone, 14.7% used DMSA alone, and 6 patients did not take any oral chelators, relying solely on periodic intravenous treatment. In terms of treatment adherence, nearly half of the patients (46.8%) felt that they adhered to regular medication completely. The majority maintained regular review (75.2%) and adhered to low-copper diets strictly (51.4%). Additionally, 85.3% had a medium level of social support, while 44.0% were in good mental health.

Liver function and copper metabolism parameters of patients at the time of admission were also collected in this study. 65.1% of patients had progressed to cirrhosis. Despite this, liver function remained relatively preserved, with 91.7% classified as Child–Pugh A and 67.0% as ALBI grade 1. Urinary copper (U-Cu) levels were generally between 100 and 1000 μg/L, though 16.5% of patients exceeded 1000 μg/L. Ceruloplasmin (CP) levels were notably low, with 96.3% below 0.1 g/L and 43.1% falling below the detection limit of our laboratory (≤ 0.02 g/L).

### Assessment of neurological symptoms in patients with WD

#### The details of UWDRS Part I scale items

UWDRS Part I consists of 27 items to assess the severity of neurological symptoms, and the internal consistency of UWDRS Part I was high featuring a Cronbach’s alpha of 0.956 in this study. Among the 84 patients with neurological symptoms, the most prevalent (present in > 50% of patients) items as defined by a score ≥ 1 were item 3 (“salivation”; 66.7%; mean = 1.23), item 10 (“speech”; 70.2%; mean = 1.36), item 15 (“finger taps”; left 75.0%, mean = 1.38; right 75.0%, mean = 1.38), item 16 (“rapid alternating movements of hands”; left 81.0%, mean = 1.45; right 76.2%, mean = 1.38), item 17 (“handwriting”; 61.9%; mean = 1.23), item 20 (“leg agility”; left 61.9%, mean = 1.13; right 60.7%, mean = 1.05), item 23 (“arm and hand dystonia”; left 56.0%, mean = 1.06; right 54.8%, mean = 1.06), item 26A (“gait – leg dystonia”; 59.5%; mean = 1.12) and item 26B (“gait – ataxia”; 58.3%; mean = 1.19) (Supplementary Table 3).

#### Sex and age differences in prevalent neurological symptoms

Ataxia gait was more prevalent and severe in females than in males, and females had a higher severity of dystonia of left arm and hand than males (all *p* < 0.05). The prevalence of the following four symptoms varied between age groups: impaired rapid alternating movements of right hand, dysarthria, dystonia of left arm and hand, dystonia of right arm and hand (all* p* < 0.05). The severity of the following 7 symptoms varied between age groups: impaired rapid alternating movements of right hand, dysarthria, salivation, impaired handwriting, impaired gait (leg dystonia), dystonia of left arm and hand, dystonia of right arm and hand (all *p* < 0.05). Generally, the prevalence and severity of these neurological symptoms were both higher in patients ≤ 20 years old than older groups (Table 1).

**Table 1:** Most prevalent neurological signs and symptoms of different sexes and ages

Symptoms	Prevalence (%)	Sex			Age (years)				
		Male (*n* = 37)	Female (*n* = 47)	*p*	≤ 20 (*n* = 7)	21–30 (*n* = 29)	31–40 (*n* = 34)	> 40 (*n* = 14)	*p*
Impaired rapid alternating movements of left hand	81.0								
*n* (%)		32 (86.5)	36 (76.6)	0.252	6 (85.7)	21 (72.4)	29 (85.3)	12 (85.7)	0.585^*^
mean score		1.41	1.49	0.992	2.00	1.28	1.59	1.21	0.250
Impaired rapid alternating movements of right hand	76.2								
*n* (%)		30 (81.1)	34 (72.3)	0.350	7 (100.0)	17 (58.6)	29 (85.3)	11 (78.6)	0.043^*^
mean score		1.32	1.43	0.859	2.14	1.03	1.59	1.21	**0.042**
Impaired left finger tapping	75.0								
*n* (%)		29 (78.4)	34 (72.3)	0.526	6 (85.7)	23 (79.3)	25 (73.5)	9 (64.3)	0.699^*^
mean score		1.41	1.36	0.765	2.00	1.34	1.44	1.00	0.306
Impaired right finger tapping	75.0								
*n* (%)		30 (81.1)	33 (70.2)	0.253	7 (100.0)	21 (72.4)	25 (73.5)	10 (71.4)	0.513^*^
mean score		1.35	1.40	0.978	2.00	1.14	1.56	1.14	0.189
Dysarthria	70.2								
*n* (%)		27 (73.0)	32 (68.1)	0.627	7 (100.0)	17 (58.6)	28 (82.4)	7 (50.0)	0.018^*^
mean score		1.46	1.28	0.507	2.86	1.00^a^	1.65	0.64^a,c^	** < 0.001**
Salivation	66.7								
*n* (%)		27 (73.0)	29 (61.7)	0.277	7 (100.0)	21 (72.4)	19 (55.9)	9 (64.3)	0.121^*^
mean score		1.35	1.13	0.285	2.43	1.24	1.15^a^	0.79^a^	**0.029**
Impaired handwriting	61.9								
*n* (%)		20 (54.1)	32 (68.1)	0.189	7 (100.0)	16 (55.2)	21 (61.8)	8 (57.1)	0.154^*^
mean score		1.16	1.28	0.596	2.43	1.00^a^	1.21	1.14	**0.029**
Impaired left leg agility	61.9								
*n* (%)		21 (56.8)	31 (66.0)	0.389	6 (85.7)	17 (58.6)	21 (61.8)	8 (57.1)	0.644^*^
mean score		0.95	1.28	0.240	1.71	1.10	1.09	1.00	0.583
Impaired right leg agility	60.7								
*n* (%)		24 (64.9)	27 (57.4)	0.490	6 (85.7)	17 (58.6)	19 (55.9)	9 (64.3)	0.568^*^
mean score		0.95	1.13	0.743	1.71	1.03	0.91	1.07	0.482
Impaired gait—leg dystonia	59.5								
*n* (%)		22 (59.5)	28 (59.6)	0.991	7 (100.0)	15 (51.7)	21 (61.8)	7 (50.0)	0.095^*^
mean score		1.03	1.19	0.620	2.43	0.86^a^	1.18^a^	0.86^a^	**0.004**
Impaired gait—ataxia	58.3								
*n* (%)		17 (45.9)	32 (68.1)	**0.041**	5 (71.4)	15 (51.7)	22 (64.7)	7 (50.0)	0.610^*^
mean score		0.84	1.47	**0.022**	2.14	0.90	1.38	0.86	0.151
Dystonia of left arm and hand	56.0								
*n* (%)		17 (45.9)	30 (63.8)	0.101	7 (100.0)	14 (48.3)	21 (61.8)	5 (35.7)^a^	0.025^*^
mean score		0.76	1.30	**0.038**	2.00	1.03	1.06	0.64^a^	**0.041**
Dystonia of right arm and hand	54.8								
*n* (%)		18 (48.6)	28 (59.6)	0.318	7 (100.0)	13 (44.8)^a^	21 (61.8)	5 (35.7)^a^	0.017^*^
mean score		0.84	1.23	0.132	2.14	0.93^a^	1.12	0.64^a^	**0.015**

^*^Fisher’s exact test

^a^*p* < 0.05 when compared with ≤ 20 years of age

^b^*p* < 0.05 when compared with 21–30 years of age

^c^*p* < 0.05 when compared with 31–40 years of age

Bold: results significant (*p* < 0.05)

### Factors associated with neurological symptoms in patients with WD

#### Association between sociodemographic/clinical characteristics and neurological symptoms

**Fig. 1:**
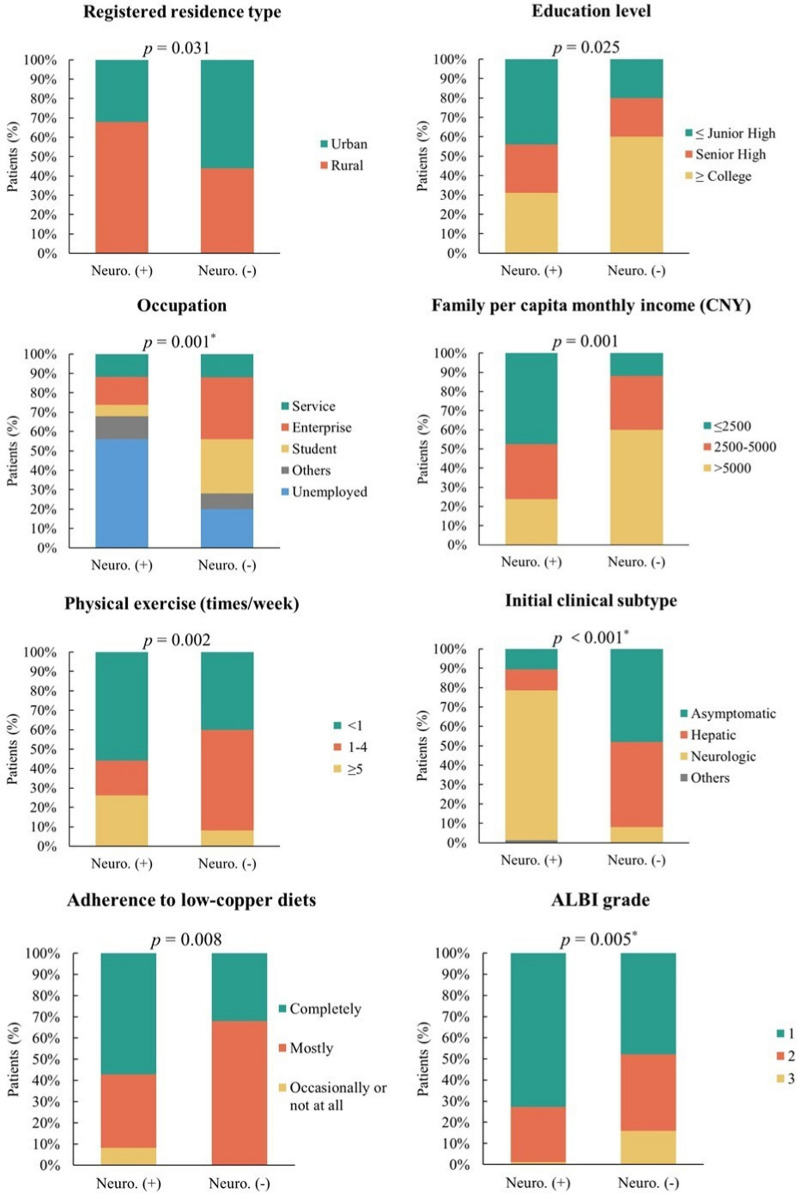
Comparison of sociodemographic and clinical characteristics between WD patients with and without neurological symptoms. Neuro. (+): with neurological symptoms, Neuro. (−): without neurological symptoms. CNY: Chinese Yuan, ALBI: Albumin-bilirubin. ^*^Fisher’s exact test

By comparing sociodemographic and clinical characteristics between patients with and without neurological symptoms, we found that patients with neurological symptoms were more likely to reside in rural areas, have lower educational attainment, be unemployed, have lower monthly per capita household income, adhere strictly to low-copper diets, and have a lower ALBI grade. They were also more likely to present with neurological symptoms at the initial onset of the disease. Only a small proportion of patients (22.6%) developed neurological symptoms later during the disease course rather than at onset. Additionally, the two groups differed in terms of physical exercise frequency (all *p* < 0.05) (Fig. 1). See Supplementary Table 2 for more details.

We further analyzed the association between patient characteristics and the severity of neurological symptoms (Supplementary Table 4). Among the 84 patients with neurological symptoms, the median score of UWDRS Part I was 30.0 (IQR: 13.3–52.0). The analysis of factors associated with the severity of neurological symptoms also included 25 patients without neurological symptoms, all of whom had a UWDRS Part I score of 0. The one-way analysis of variance showed that UWDRS Part I scores were significantly different among groups with distinct levels of nine factors (registered residence type, BMI, educational level, occupation, family per capita monthly income, initial clinical subtype, adherence to low-copper diets, mental health conditions, and ALBI grade, all *p* < 0.05). Patients who were rural residents, had a lower BMI, received less education, were unemployed, had a lower monthly family income, presented neurological symptoms at disease onset, adhered poorly to low-copper diets, had worse mental health, and a lower ALBI grade were more likely to have higher scores (Table 2).

**Table 2:** Comparison of UWDRS Part I scores across sociodemographic and clinical characteristics in WD patients, *M* (*Q*_1_, *Q*_3_)

Characteristics	Score	*p*
*Registered residence type*		
Urban	13.0 (0.0, 29.5)	**0.013**
Rural	27.0 (6.3, 51.8)	
*BMI (kg/m*^*2*^*)*		
< 18.5	35.0 (11.5, 53.0)	**0.009**
18.5–24	16.0 (3.3, 40.5)	
≥ 24	7.0 (0.0, 20.0)	
*Education level*		
Junior high school and below	29.5 (6.5, 51.3)	**0.001**
Senior high school	34.5 (10.8, 69.0)	
College and above	6.0 (0.0, 21.0)	
*Occupation*		
Personnel of service industries	14.0 (1.0, 19.0)	** < 0.001**
Personnel of enterprises or institutions	5.0 (0.0, 16.8)	
Student	0.0 (0.0, 55.3)	
Others	12.5 (3.5, 18.8)	
Unemployed	35.5 (17.5, 61.5)	
*Family per capita monthly income (CNY)*		
≤ 2500	33.0 (13.0, 60.0)	** < 0.001**
2500–5000	20.0 (5.0, 47.0)	
> 5000	4.0 (0.0, 16.0)	
*Initial clinical subtype*		
Neurologic	29.0 (15.0, 52.0)	** < 0.001**
Non-neurologic	0.0 (0.0, 22.5)	
*Adherence to low-copper diets*		
Completely	28.0 (8.5, 53.0)	**0.001**
Mostly	6.0 (0.0, 26.3)	
Occasionally or not at all	45.0 (6.0, 100.0)	
*Mental health*		
Good	13.0 (1.3, 28.0)	**0.004**
Moderate	28.5 (0.0, 51.3)	
Poor	22.0 (2.0, 62.5)	
Bad	57.5 (32.5, 101.5)	
*ALBI grade*		
1	20.0 (4.5, 51.0)	**0.031**
2	20.0 (0.0, 35.0)	
3	0.0 (0.0, 9.5)	

All the examined continuous variables failed to conform to the normal distribution, thus results were presented as median (interquartile range)

BMI, Body mass index; CNY, Chinese Yuan; ALBI, Albumin-bilirubin

Bold: results significant (*p* < 0.05)

UWDRS Part I scores were significantly different among groups with distinct levels of nine factors, which were included as independent variables in the regression analysis. The stepwise multiple linear regression analysis showed that factors associated with UWDRS Part I scores among WD patients were occupation, family per capita monthly income, initial clinical subtype, adherence to low-copper diets, and mental health conditions (all *p* < 0.05), and the explanatory power of these factors was 42.1% (*F* = 10.474, *p* < 0.001). There was no significant colinearity among the variables (VIF = 1.040–1.190). UWDRS Part I scores were higher among patients who were unemployed, had a lower monthly family income, presented neurological symptoms at disease onset, adhered poorly to low-copper diets, and had worse mental health (Table 3). The intermediate iteration process is shown in Supplementary Table 5.

**Table 3:** Multiple linear regression analysis of factors associated with UWDRS Part I scores

Variable	*β*	95% *CI*	*SE*	*t*	*p*
*Occupation (ref: unemployed)*					
Personnel of service industries	− 23.415	− 38.127, − 8.703	7.506	− 3.119	**0.002**
Personnel of enterprises or institutions	− 23.496	− 36.364, − 10.628	6.566	− 3.579	**0.001**
Others	− 16.921	− 32.595, − 1.247	7.997	− 2.116	**0.037**
*Family per capita monthly income (ref:* ≤ *2500)*					
> 5000	− 11.107	− 21.847, − 0.366	5.480	− 2.027	**0.045**
Initial clinical subtype (ref: neurologic)					
Non-neurologic	− 12.154	− 22.070, − 2.238	5.059	− 2.402	**0.018**
*Adherence to low-copper diets (ref: occasionally or not at all)*					
Mostly	− 14.673	− 24.459, − 4.888	4.993	− 2.939	**0.004**
Mental health (ref: good)					
Bad	27.621	7.061, 48.180	10.490	2.633	**0.010**

CI, Confidence interval; SE, Standard error

Bold: results significant (*p* < 0.05)

#### Association between ATP7B genotypes and neurological symptoms

**Fig. 2:**
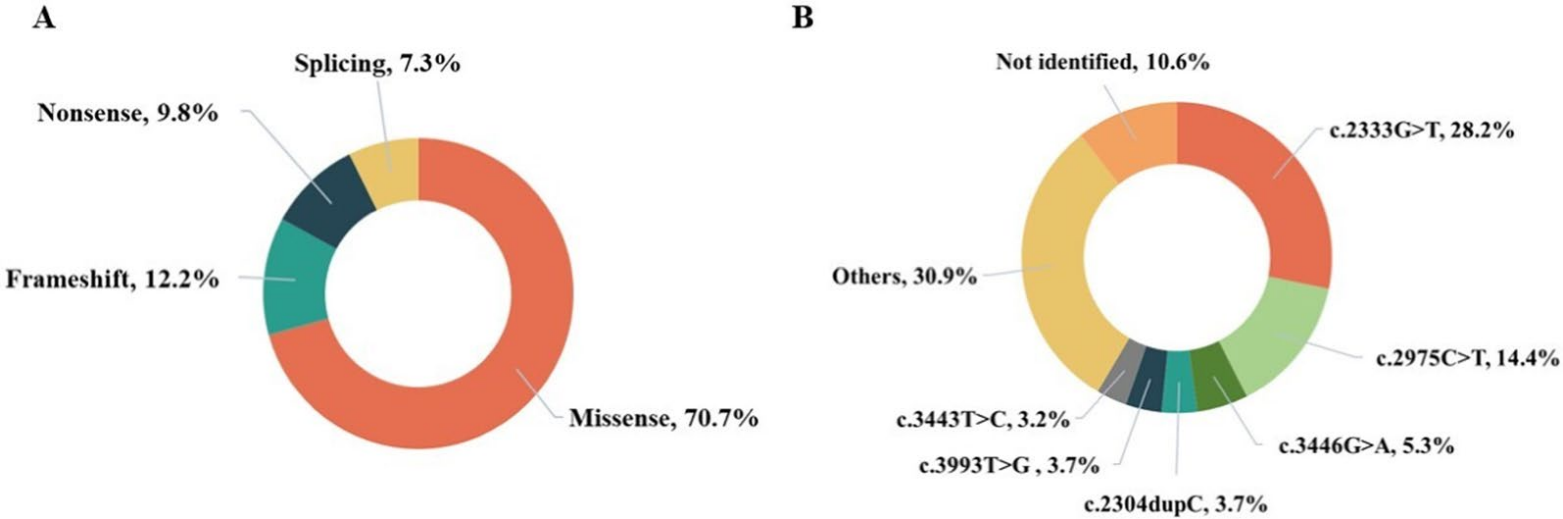
ATP7B gene mutations. **A** Distribution of mutation types, **B** Allele frequencies of common variants

Among the 109 patients investigated, whole-genome resequencing was performed on 94 patients from 94 unrelated families. 76 patients (80.9%) exhibited pathogenic mutations in both alleles, including 17 homozygotes and 59 compound heterozygotes. A total of 41 distinct pathogenic mutations were identified, including 29 missense mutations, 5 frameshift mutations, 4 nonsense mutations, and 3 splice-site mutations (Fig. 2A). The most frequent mutation was c.2333G > T (p.R778L) in exon 8, with an allelic frequency of 28.2% (53/188), followed by c.2975C > T (p.P992L) in exon 13 (14.4%, 27/188) (Fig. 2B). Additionally, 59 different pathogenic mutation combinations were observed. The most prevalent genotype was homozygous c.2333G > T (p.R778L/p.R778L), present in 8.5% (8/94) of patients, followed by the compound heterozygous genotype c.2333G > T/c.2975C > T (p.R778L/p.P992L), also observed in 8.5% (8/94).

**Fig. 3:**
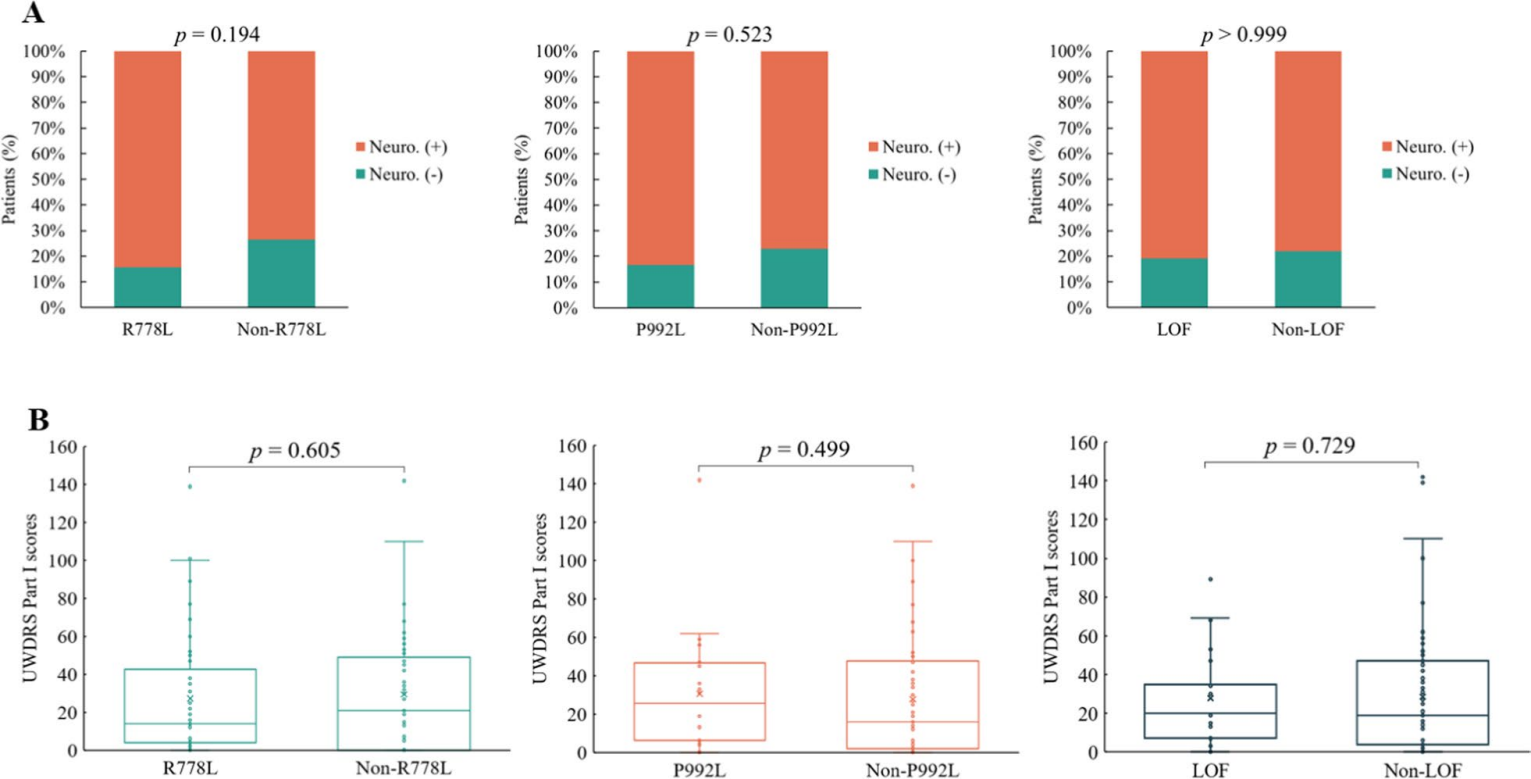
Comparison of neurological phenotype among different genotypes. **A** Presence of neurological symptoms, **B** UWDRS Part I scores. Neuro. (+): with neurological symptoms, Neuro. (−): without neurological symptoms. UWDRS, Unified Wilson’s Disease Rating Scale, LOF: Loss-of-function

Among the 94 unrelated patients, 74 (78.7%) presented with neurological symptoms. The median UWDRS Part I score for these patients was 19.0 (IQR: 3.8–47.0). We analyzed the associations of the two most common mutations (p.R778L and p.P992L) and LOF mutations with the presence of neurological symptoms and UWDRS Part I scores (Fig. 3). No significant associations were identified. Additionally, we examined the prevalence of common neurological symptoms across different genotypes. Patients carrying the p.P992L mutation showed a significantly higher frequency of impaired finger tapping (*p* = 0.037), while no other neurological symptoms were found to be genotype-associated (Table 4).

**Table 4:** Prevalence of common neurological symptoms at enrollment across different genotypes, *n* (%)

	LOF mutations			R778L mutation			P992L mutation		
	Yes	No	*p*	Yes	No	*p*	Yes	No	*p*
Impaired hand rapid alternating movements	15	48	0.626	30	33	0.944	17	46	0.645
	(71.4)	(65.8)		(66.7)	(67.3)		(70.8)	(65.7)	
Impaired finger tapping	14	48	0.938	27	35	0.243	20	42	**0.037**
	(66.7)	(65.8)		(60.0)	(71.4)		(83.3)	(60.0)	
Dysarthria	15	37	0.092	22	30	0.229	16	36	0.195
	(71.4)	(50.7)		(48.9)	(61.2)		(66.7)	(51.4)	
Salivation	10	46	0.205	28	28	0.616	16	40	0.412
	(47.6)	(63.0)		(62.2)	(57.1)		(66.7)	(57.1)	
Impaired handwriting	12	35	0.458	21	26	0.536	12	35	> 0.999
	(57.1)	(47.9)		(46.7)	(53.1)		(50.0)	(50.0)	

LOF, Loss of function

Bold: results significant (*p* < 0.05)

## Discussion

Neurological symptoms occur in almost half of WD patients and vary in both type and severity of presentation [1, 8]. Among the 109 patients included in this study, 84 (77.1%) had neurological symptoms, and the neurological manifestations of patients were assessed using UWDRS Part I (the neurological subscale of the UWDRS). The most common neurological sign items included impaired rapid alternating movements of hands, impaired finger tapping, dysarthria, salivation, impaired handwriting, impaired leg agility, ataxia gait, and dystonia of the limbs, which is very similar to the previous reports [8, 10]. However, the occurrence of tremor in the current study is lower than that in a previous study conducted in the Polish cohort [8]. The discrepancies could be explained by the fact that patients in our study had received prior treatment, whereas those in the Polish study were newly diagnosed and untreated. And Burke JF et al. demonstrated that tremor was less refractory and might be associated with greater improvements with duration of treatment [26].

There were sex and age differences in the prevalence and severity of neurological symptoms in this study. Ataxia gait was more prevalent and severe in females. In line with previous studies on sex differences in Parkinson’s disease, females with Parkinson’s disease also exhibited greater postural instability and scored worse on the Unified Parkinson’s Disease Rating Scale (UPDRS) for instability compared to males [27, 28]. Generally, neurological signs and symptoms were more common and more severe in patients 20 years old and younger. A possible explanation is that neurological symptoms may persist for longer periods of time and improvement may only be observed after years of treatment [7]. In some cases, neurological symptoms can even worsen at the initial stage of treatment [29–31].

Notably, this study showed that sociodemographic and clinical factors associated with the severity of neurological symptoms of WD patients were occupation, family per capita monthly income, initial clinical subtype, adherence to low-copper diets, and mental health conditions. Firstly, in our study, 47.7% of WD patients were unemployed, and 39.4% had a monthly per capita household income below CNY2500. Neurological manifestations of WD significantly impair patients’ ability to work, leading to long-term unemployment and financial dependence on family members or social welfare programmes. As reported by Masełbas W et al., only 19.6% of WD patients with neurological symptoms had a salary as their main source of income [32], highlighting the substantial socioeconomic burden associated with WD. Secondly, patients who did not exhibit neurological symptoms at disease onset tend to have milder neurological impairment later in the disease course. Early initiation of copper chelation therapy, before neurological symptoms appear, may be effective to prevent or delay their onset, helping to preserve neurological function. In contrast, patients who initially presented with neurological symptoms not only experience persistent dysfunction but also tend to more severe and disabling manifestations. Thirdly, we also observed worse neurological symptoms in patients with poorer adherence to low-copper diets. Dietary copper restriction has long been recognized as an important aspect of the treatment of WD, as reducing dietary copper intake may help slow disease progression. However, in this study, patients who were able to adhere to low-copper diets most of the time but occasionally failed to do so appeared to have lower UWDRS Part I scores than those who restricted their diet completely. This finding suggests that further thought should be given to the rationale for long-term dietary copper restriction on WD patients. Based on current evidence, adherence to medication is more important than dietary copper restriction for WD patients, and the only two foods that should consistently be avoided are liver and shellfish [33, 34]. Lastly, only 44.0% of patients were in good mental health in this study. A possible explanation is that depression and anxiety may be triggered by long-term treatment, repeated hospitalizations, low-copper diets, high treatment costs, and movement disorders [35]. And Wilson’s disease itself may present psychiatric symptoms of anxiety and depression [1, 4]. Furthermore, multivariate linear regression analysis revealed that psychological distress was associated with the severity of neurological disease. It is reported that stress is one of the factors which may aggravate symptoms of WD [7, 36]. Psychological distress can exacerbate the conditions of dystonia, tremor, salivation and dysarthria, and the more severe the symptoms, the more stressed the patient will be, thus entering into a vicious circle.

Additionally, we analyzed the association between genotype and neurological phenotype in 94 unrelated patients and found no significant association between genotype and either the presence or severity of neurological symptoms. However, when analyzing specific neurological symptoms, we observed that patients carrying the p.P992L mutation had a significantly higher frequency of impaired finger tapping. In contrast, no significant association was observed between this mutation and other fine motor symptoms, such as impaired handwriting. While these findings may indicate a potential association between p.P992L and specific neurological symptoms, current evidence regarding the relationship between this mutation and neurological phenotypes remains scarce and warrants further investigation.

This study has several limitations. WD is a rare disease, and our study is based on a relatively small group of patients. Secondly, the neurological symptoms of WD are intricate and varied, which may make it difficult to distinguish one symptom from another [26]. Additionally, as a cross-sectional study, it cannot establish causal relationships between the factors and the severity of symptoms but can only suggest potential correlations. Despite these constraints, our results may still provide valuable insights. In the future, we aim to conduct longitudinal and more in-depth studies to better explore the factors influencing the neurological symptoms.

## Conclusion

In summary, the main neurological symptoms of patients with WD were lack of motor coordination, dystonia, dysarthria, and salivation. We observed sex and age differences in the neurological features of WD. Occupation, family per capita monthly income, initial clinical subtype, adherence to low-copper diets and mental health conditions were associated with the severity of neurological symptoms in WD. Patients carrying the p.P992L mutation had a significantly higher frequency of impaired finger tapping. This study evaluated the neurological signs and symptoms of WD patients in Southwest China using the UWDRS and explored factors associated with neurological symptoms. These findings may further deepen the understanding of WD and guide future management of patients to improve prognosis.

## Supplementary Information

Additional file1

Additional file2

Additional file3

Additional file4

Additional file5

## Data Availability

The data underlying this article will be shared on reasonable request to the corresponding author.
