# Supplementary material for: Assessment of neurological symptoms and associated factors in patients with Wilson’s disease in Southwest China
Source: Orphanet J Rare Dis. 2025 Jul 4;20:342. doi: 10.1186/s13023-025-03874-2 (PMC12228280; doi:10.1186/s13023-025-03874-2)
Supplement: Supplementary file 1 — Additional file1 [file 13023_2025_3874_MOESM1_ESM.docx]

**Supplementary Table 1** Questionnaire for patients with Wilson’s disease

| Questionnaire for patients with Wilson’s disease | |
| --- | --- |
| We invite you to participate in this survey aimed at better understanding Wilson’s disease. Your input will help us gather valuable insights to develop strategic recommendations for the diagnosis and treatment of different populations and to provide better guidance for the prognosis of patients. This survey is anonymous, and we will keep your personal information confidential. Please fill out the survey truthfully based on your actual situation. | |
| Section 1: Demographic Information | |
| 1.1 Sex:  🞏 Male 🞏 Female | |
| 1.2 Date of Birth: [MM/DD/YYYY] | |
| 1.3 Registered residence type :  🞏 Rural 🞏 Urban | |
| 1.4 Height: _______ cm | |
| 1.5 Weight: _______ kg | |
| 1.6 Education Level:  🞏 Primary school and below 🞏 Junior high school  🞏 Senior high school 🞏 College and above | |
| 1.7 Marital status :  🞏 Unmarried 🞏 Married 🞏 Divorced 🞏 Widowed | |
| 1.8 Occupation :  🞏 Personnel of service industries 🞏 Personnel of enterprises or institutions  🞏 Student 🞏 Unemployed 🞏 Other (please specify): ___________ | |
| 1.9 What is your monthly per capita household income? (RMB)  🞏 ≤2500 🞏 2500-5000 🞏 5000-10000 🞏 >10000 | |
| Section 2: Lifestyle Habits | |
| 2.1 In the past six months, how often have you engaged in physical exercise or sports?  🞏 <1 times/week 🞏 1-2 times/week 🞏 3-4 times/week 🞏 ≥5 times/week | |
| 2.2 In the past six months, how many hours do you sleep on average each day?  🞏 <6 hours 🞏 6-8 hours 🞏 8-10 hours 🞏 ≥10 hours | |
| 2.3 In the past six months, how do you feel about the quality of your sleep?  🞏 Very good 🞏 Good 🞏 Moderate 🞏 Poor 🞏 Very poor | |
| 2.4 In the past six months, what is your smoking status?  🞏 Regular smoker 🞏 Occasional smoker 🞏 Quit smoking 🞏 Never smoked | |
| 2.5 In the past six months, how often do you consume alcohol?  🞏 Every week 🞏 Less than once a week, but more than once a month  🞏 Only during certain seasons/occasions 🞏 Occasionally 🞏 Never | |
| Section 3: Medical History | |
| 3.1 Do you have any family history of Wilson’s disease?  🞏 Yes (please specify your relationship to the relative with Wilson’s disease):  🞏 No | |
| 3.2 Have you experienced any symptoms related to Wilson’s disease?  🞏 Always asymptomatic  🞏 First symptoms (please specify the symptoms and the time of onset) | |
| 3.3 Date of your diagnosis of Wilson’s disease: [MM/DD/YYYY] | |
| 3.4 Have you been diagnosed with any other medical conditions?  🞏 Yes (please specify): _____________ 🞏 No | |
| Section 4: Treatment-related information | |
| 4.1 Date of initiation of initial treatment after diagnosis of Wilson’s disease: [MM/DD/YYYY] | |
| 4.2 What copper chelation therapy regimen are you currently using? (please specify the dosage and course of treatment)  🞏 DPA 🞏 DMSA 🞏 DMPS 🞏Zn  🞏 DPA+ Zn 🞏 DMPS+ Zn 🞏 DMSA+Zn 🞏 DMPS+ DMSA  🞏 DPA+ DMPS 🞏 DPA+ DMSA 🞏 DPA+ DMPS+ Zn  🞏 DPA+ DMSA+ Zn 🞏 DMPS+ DMSA+ Zn 🞏 DPA+ DMPS+ DMSA + Zn  🞏 Others (please specify): _____________ | |
| 4.3 Do you take your medication regularly?  🞏 Completely 🞏 Mostly 🞏 Occasionally 🞏 Not at all | |
| 4.4 Do you attend regular medical reviews?  🞏 Completely 🞏 Mostly 🞏 Occasionally 🞏 Not at all | |
| 4.5 Do you adhere to a low-copper diet?  🞏 Completely 🞏 Mostly 🞏 Occasionally 🞏 Not at all | |
| Section 5: Social Support (the Social Support Rating Scale, SSRS)  *The SSRS is a self-report questionnaire in Chinese to measure objective support (three items), subjective support (four items), and support use (three items). This scale consists of 10 items, with a total score ranging from 12 to 66 points. The higher the score, the more the social support.* | |
| 5.1 How many close friends do you have?  🞏 None 🞏 1-2 🞏 3-5 🞏 6 or more than 6 | |
| 5.2 In the past year…… ?  🞏 I lived alone, away from home  🞏 I moved from place to place, and met different neighbors or roommates  🞏 I lived on campus, or with coworkers or friends  🞏 I lived with my family | |
| 5.3 The relationship between you and your neighbor is…… ?  🞏 Nodding acquaintance  🞏 Concerned occasionally  🞏 Some neighbors care about me  🞏 Most of the neighbors care about me | |
| 5.4 The relationship between you and your coworkers is…… ?  🞏 Nodding acquaintance  🞏 Concerned occasionally  🞏 Some coworkers care about me  🞏 Most of the coworkers care about me | |
| 5.5 Support and help from family members | |
| A. Spouse/ loved ones  B. Parents  C. Sons and daughters  D. Sisters and brothers  E. Other relatives | 🞏No 🞏Rarely 🞏 So so 🞏Fully support  🞏No 🞏Rarely 🞏 So so 🞏Fully support  🞏No 🞏Rarely 🞏 So so 🞏Fully support  🞏No 🞏Rarely 🞏 So so 🞏Fully support  🞏No 🞏Rarely 🞏 So so 🞏Fully support |
| 5.6 The recourses where you got financial and solid support when you were in need of help?  🞏 Nobody  🞏 The following (more than one choice could be selected):  A. spouse; B. other family members; C. friends; D. relatives; E.co-worker; F. work union; G. official authorities; H. nonofficial groups I. other (please list below) | |
| 5.7 The resources where you got console and care when you were in need of help?  🞏 Nobody  🞏 The following (more than one choice could be selected):  A. spouse; B. other family members; C. friends; D. relatives; E.co-worker; F. work union; G. Official authorities; H. nonofficial groups; I. other (please list below) | |
| 5.8 Who do you express yourself when in trouble?  🞏 I never tell anyone  🞏 I share with most intimate 1or 2 friends  🞏 I share with friends who concerns  🞏 I vent it, and get help from others | |
| 5.9 How do you get help when in need?  🞏 I am self-dependent  🞏 I seldom ask for help from others  🞏 I sometimes ask for help from others  🞏 I often ask for help from family relatives and friends | |
| 5.10 How often do you take part in the social groups (party activities, religion groups or student union)?  🞏 Never 🞏 Seldom 🞏 Sometimes 🞏 An active member | |
| Section 6: Mental Health (the Kessler Psychological Distress Scale, K10)  *The K10 is a 10-item self-report questionnaire using a Likert-type scale, with a total score ranging from 10 to 50 points. The lower the score, the greater the level of mental health conditions.* | |
| 6.1 During the last 30 days, about how often did you feel tired out for no good reason?  🞏 None of the time 🞏 A little of the time 🞏 Some of the time  🞏 Most of the time 🞏 All of the time | |
| 6.2 During the last 30 days, about how often did you feel nervous?  🞏 None of the time 🞏 A little of the time 🞏 Some of the time  🞏 Most of the time 🞏 All of the time | |
| 6.3 During the last 30 days, about how often did you feel so nervous that nothing could calm you down?  🞏 None of the time 🞏 A little of the time 🞏 Some of the time  🞏 Most of the time 🞏 All of the time | |
| 6.4 During the last 30 days, about how often did you feel hopeless?  🞏 None of the time 🞏 A little of the time 🞏 Some of the time  🞏 Most of the time 🞏 All of the time | |
| 6.5 During the last 30 days, about how often did you feel restless or fidgety?  🞏 None of the time 🞏 A little of the time 🞏 Some of the time  🞏 Most of the time 🞏 All of the time | |
| 6.6 During the last 30 days, about how often did you feel so restless you could not sit still?  🞏 None of the time 🞏 A little of the time 🞏 Some of the time  🞏 Most of the time 🞏 All of the time | |
| 6.7 During the last 30 days, about how often did you feel depressed?  🞏 None of the time 🞏 A little of the time 🞏 Some of the time  🞏 Most of the time 🞏 All of the time | |
| 6.8 During the last 30 days, about how often did you feel that everything was an effort?  🞏 None of the time 🞏 A little of the time 🞏 Some of the time  🞏 Most of the time 🞏 All of the time | |
| 6.9 During the last 30 days, about how often did you feel so sad that nothing could cheer you up?  🞏 None of the time 🞏 A little of the time 🞏 Some of the time  🞏 Most of the time 🞏 All of the time | |
| 6.10 During the last 30 days, about how often did you feel worthless?  🞏 None of the time 🞏 A little of the time 🞏 Some of the time  🞏 Most of the time 🞏 All of the time | |
| This is the end of the questionnaire. Thank you for your participation. | |
