# Supplementary material for: Assessment of neurological symptoms and associated factors in patients with Wilson’s disease in Southwest China
Source: Orphanet J Rare Dis. 2025 Jul 4;20:342. doi: 10.1186/s13023-025-03874-2 (PMC12228280; doi:10.1186/s13023-025-03874-2)
Supplement: Supplementary file 2 — Additional file2 [file 13023_2025_3874_MOESM2_ESM.docx]

**Supplementary Table 2** Sociodemographic and clinical characteristics of patients with WD, *n* (%).

| **Characteristics** | **Total**  **(*n* = 109)** | **With neurological symptoms**  **(*n* = 84)** | **Without neurological symptoms**  **(*n* = 25)** | ***p*** |
| --- | --- | --- | --- | --- |
| **Demographic Characteristics** |  |  |  |  |
| Sex |  |  |  |  |
| Male | 48 (44.0) | 37 (44.0) | 11 (44.0) | 0.997 |
| Female | 61 (56.0) | 47 (56.0) | 14 (56.0) |  |
| Age (years) |  |  |  |  |
| ≤20 | 13 (11.9) | 7 (8.3) | 6 (24.0) | 0.090^*^ |
| 21-30 | 40 (36.7) | 29 (34.5) | 11 (44.0) |  |
| 31-40 | 40 (36.7) | 34 (40.5) | 6 (24.0) |  |
| >40 | 16 (14.7) | 14 (16.7) | 2 (8.0) |  |
| Registered residence type |  |  |  |  |
| Urban | 41 (37.6) | 27 (32.1) | 14 (56.0) | **0.031** |
| Rural | 68 (62.4) | 57 (67.9) | 11 (44.0) |  |
| BMI (kg/m^2^) |  |  |  |  |
| <18.5 | 30 (27.5) | 25 (29.8) | 5 (20.0) | 0.086 |
| 18.5-24 | 60 (55.0) | 48 (57.1) | 12 (48.0) |  |
| ≥24 | 19 (17.4) | 11 (13.1) | 8 (32.0) |  |
| Education level |  |  |  |  |
| Junior high school and below | 42 (38.5) | 37 (44.0) | 5 (20.0) | **0.025** |
| Senior high school | 26 (23.9) | 21 (25.0) | 5 (20.0) |  |
| College and above | 41 (37.6) | 26 (31.0) | 15 (60.0) |  |
| Marital status |  |  |  |  |
| Unmarried | 50 (45.9) | 34 (40.5) | 16 (64.0) | 0.053 |
| Married | 49 (45.0) | 40 (47.6) | 9 (36.0) |  |
| Divorced or widowed | 10 (9.2) | 10 (11.9) | 0 (0.0) |  |
| Occupation |  |  |  |  |
| Personnel of service industries | 13 (11.9) | 10 (11.9) | 3 (12.0) | **0.001^*^** |
| Personnel of enterprises or institutions | 20 (18.3) | 12 (14.3) | 8 (32.0) |  |
| Student | 12 (11.0) | 5 (6.0) | 7 (28.0) |  |
| Others | 12 (11.0) | 10 (11.9) | 2 (8.0) |  |
| Unemployed | 52 (47.7) | 47 (56.0) | 5 (20.0) |  |
| Family per capita monthly income (CNY) |  |  |  |  |
| ≤2500 | 43 (39.4) | 40 (47.6) | 3 (12.0) | **0.001** |
| 2500-5000 | 31 (28.4) | 24 (28.6) | 7 (28.0) |  |
| >5000 | 35 (32.1) | 20 (23.8) | 15 (60.0) |  |
| **Lifestyle Habits** |  |  |  |  |
| Physical exercise (times/week) |  |  |  |  |
| <1 | 57 (52.3) | 47 (56.0) | 10 (40.0) | **0.002** |
| 1-4 | 28 (25.7) | 15 (17.9) | 13 (52.0) |  |
| ≥5 | 24 (22.0) | 22 (26.2) | 2 (8.0) |  |
| Sleep duration (hours) |  |  |  |  |
| <6 | 18 (16.5) | 17 (20.2) | 1 (4.0) | 0.092 |
| 6-7 | 57 (52.3) | 44 (52.4) | 13 (52.0) |  |
| ≥8 | 34 (31.2) | 23 (27.4) | 11 (44.0) |  |
| Sleep quality |  |  |  |  |
| Good | 60 (55.0) | 45 (53.6) | 15 (60.0) | 0.092 |
| Moderate | 24 (22.0) | 16 (19.0) | 8 (32.0) |  |
| Poor | 25 (22.9) | 23 (27.4) | 2 (8.0) |  |
| Smoking status |  |  |  |  |
| Yes | 10 (9.2) | 8 (9.5) | 2 (8.0) | >0.999 |
| No | 99 (90.8) | 76 (90.5) | 23 (92.0) |  |
| Drinking status |  |  |  |  |
| Yes | 14 (12.8) | 12 (14.3) | 2 (8.0) | 0.628 |
| No | 95 (87.2) | 72 (85.7) | 23 (92.0) |  |
| **Disease-related Information** |  |  |  |  |
| Positive family history |  |  |  |  |
| Yes | 28 (25.7) | 24 (28.6) | 4 (16.0) | 0.207 |
| No | 81 (74.3) | 60 (71.4) | 21 (84.0) |  |
| Age at onset (years) |  |  |  |  |
| ≤15 | 33 (30.3) | 22 (26.2) | 11 (44.0) | 0.329 |
| 16-20 | 34 (31.2) | 28 (33.3) | 6 (24.0) |  |
| 21-25 | 16 (14.7) | 12 (14.3) | 4 (16.0) |  |
| >25 | 26 (23.9) | 22 (26.2)) | 4 (16.0) |  |
| Initial clinical subtype |  |  |  |  |
| Asymptomatic | 21 (19.3) | 9 (10.7) | 12 (48.0) | **<0.001****^*^** |
| Hepatic | 20 (18.3) | 9 (10.7) | 11 (44.0) |  |
| Neurologic | 67 (61.5) | 65 (77.4) | 2 (8.0) |  |
| Others | 1 (0.9) | 1 (1.2) | 0 (0.0) |  |
| Onset to diagnosis (years) |  |  |  |  |
| <1 | 75 (68.8) | 54 (64.3) | 21 (84.0) | 0.133**^*^** |
| 1-2 | 17 (15.6) | 16 (19.0) | 1 (4.0) |  |
| >2 | 17 (15.6) | 14 (16.7) | 3 (12.0) |  |
| Misdiagnosed |  |  |  |  |
| Yes | 59 (54.1) | 43 (51.2) | 16 (64.0) | 0.259 |
| No | 50 (45.9) | 41 (48.8) | 9 (36.0) |  |
| Disease duration (years) |  |  |  |  |
| ≤5 | 30 (27.5) | 19 (22.6) | 11 (44.0) | 0.291**^*^** |
| 6-10 | 29 (26.6) | 22 (26.2) | 7 (28.0) |  |
| 11-15 | 27 (24.8) | 23 (27.4) | 4 (16.0) |  |
| 16-20 | 14 (12.8) | 12 (14.3) | 2 (8.0) |  |
| >20 | 9 (8.3) | 8 (9.5) | 1 (4.0) |  |
| Years of treatment (years) |  |  |  |  |
| ≤5 | 45 (41.3) | 32 (38.1) | 13 (52.0) | 0.448**^*^** |
| 6-10 | 20 (18.3) | 14 (16.7) | 6 (24.0) |  |
| 11-15 | 25 (22.9) | 22 (26.2) | 3 (12.0) |  |
| 16-20 | 10 (9.2) | 8 (9.5) | 2 (8.0) |  |
| >20 | 9 (8.3) | 8 (9.5) | 1 (4.0) |  |
| **Treatment and Adherence** |  |  |  |  |
| Intravenous chelating agent |  |  |  |  |
| DMPS | 99 (90.8) | 78 (92.9) | 21 (84.0) | 0.341 |
| EDTA | 10 (9.2) | 6 (7.1) | 4 (16.0) |  |
| Oral chelating agent |  |  |  | 0.715**^*^** |
| DPA | 18 (16.5) | 16 (19.0) | 2 (8.0) |  |
| DMSA | 16 (14.7) | 11 (13.1) | 5 (20.0) |  |
| Zn | 2 (1.8) | 2 (2.4) | 0 (0.0) |  |
| DPA+DMSA | 6 (5.5) | 5 (6.0) | 1 (4.0) |  |
| DPA+Zn | 23 (21.1) | 18 (21.4) | 5 (20.0) |  |
| DPA+DMSA+Zn | 9 (8.3) | 8 (9.5) | 1 (4.0) |  |
| DMSA+Zn | 29 (26.6) | 20 (23.8) | 9 (36.0) |  |
| None | 6 (5.5) | 4 (4.8) | 2 (8.0) |  |
| Regular medication |  |  |  |  |
| Completely | 51 (46.8) | 43 (51.2) | 8 (32.0) | 0.220 |
| Mostly | 33 (30.3) | 24 (28.6) | 9 (36.0) |  |
| Occasionally or not at all | 25 (22.9) | 17 (20.2) | 8 (32.0) |  |
| Regular review |  |  |  |  |
| Completely | 82 (75.2) | 60 (71.4) | 22 (88.0) | 0.151**^*^** |
| Mostly | 17 (15.6) | 14 (16.7) | 3 (12.0) |  |
| Occasionally or not at all | 10 (9.2) | 10 (11.9) | 0 (0.0) |  |
| Adherence to low-copper diets |  |  |  |  |
| Completely | 56 (51.4) | 48 (57.1) | 8 (32.0) | **0.008** |
| Mostly | 46 (42.2) | 29 (34.5) | 17 (68.0) |  |
| Occasionally or not at all | 7 (6.4) | 7 (8.3) | 0 (0.0) |  |
| **Psychosocial Factors** |  |  |  |  |
| Social support |  |  |  |  |
| High | 13 (11.9) | 8 (9.5) | 5 (20.0) | 0.282**^*^** |
| Medium | 93 (85.3) | 73 (86.9) | 20 (80.0) |  |
| Low | 3 (2.8) | 3 (3.6) | 0 (0.0) |  |
| Mental health |  |  |  |  |
| Good | 48 (44.0) | 37 (44.0) | 11 (44.0) | 0.669**^*^** |
| Moderate | 34 (31.2) | 25 (29.8) | 9 (36.0) |  |
| Poor | 21 (19.3) | 16 (19.0) | 5 (20.0) |  |
| Bad | 6 (5.5) | 6 (7.1) | 0 (0.0) |  |
| **Liver Function** |  |  |  |  |
| Cirrhosis |  |  |  |  |
| Yes | 71 (65.1) | 54 (64.3) | 17 (68.0) | 0.732 |
| No | 38 (34.9) | 30 (35.7) | 8 (32.0) |  |
| Child-Pugh class |  |  |  |  |
| A | 100 (91.7) | 79 (94.0) | 21 (84.0) | 0.157**^*^** |
| B | 4 (3.7) | 2 (2.4) | 2 (8.0) |  |
| C | 5 (4.6) | 3 (3.6) | 2 (8.0) |  |
| ALBI grade |  |  |  |  |
| 1 | 73 (67.0) | 61 (72.6) | 12 (48.0) | **0.005^*^** |
| 2 | 31 (28.4) | 22 (26.2) | 9 (36.0) |  |
| 3 | 5 (4.6) | 1 (1.2) | 4 (16.0) |  |
| **Copper Metabolism** |  |  |  |  |
| U-Cu (μg/L) |  |  |  |  |
| ≤100 | 20 (18.3) | 17 (20.2) | 3 (12.0) | 0.601**^*^** |
| 100-200 | 24 (22.0) | 18 (21.4) | 6 (24.0) |  |
| 200-500 | 25 (22.9) | 21 (25.0) | 4 (16.0) |  |
| 500-1000 | 22 (20.2) | 16 (19.0) | 6 (24.0) |  |
| >1000 | 18 (16.5) | 12 (14.3) | 6 (24.0) |  |
| CP (g/L) |  |  |  |  |
| ≤0.02 | 47 (43.1) | 37 (44.0) | 10 (40.0) | 0.974**^*^** |
| 0.02-0.06 | 50 (45.9) | 38 (45.2) | 12 (48.0) |  |
| 0.06-0.1 | 8 (7.3) | 6 (7.1) | 2 (8.0) |  |
| >0.1 | 4 (3.7) | 3 (3.6) | 1 (4.0) |  |

BMI, body mass index; CNY, Chinese Yuan; DMPS, sodium dimercaptopropanesulfonate; EDTA, calcium sodium edetate; DPA, D-penicillamine; DMSA, dimercaptosuccinic acid; Zn, zinc; ALBI, albumin-bilirubin; U-Cu, urinary copper; CP, ceruloplasmin.

^*^Fisher’s exact test

Bold: results significant (*p*＜0.05).
