# Supplementary material for: Assessment of neurological symptoms and associated factors in patients with Wilson’s disease in Southwest China
Source: Orphanet J Rare Dis. 2025 Jul 4;20:342. doi: 10.1186/s13023-025-03874-2 (PMC12228280; doi:10.1186/s13023-025-03874-2)
Supplement: Supplementary file 3 — Additional file3 [file 13023_2025_3874_MOESM3_ESM.docx]

**Supplementary Table 3** Item evaluation of UWDRS Part I scale.

| **Item (no.)** | ***n* (%)** | **Mean score per item** |
| --- | --- | --- |
| Mobility (1) | 33 (39.3) | 0.54 |
| Falling (2) | 20 (23.8) | 0.48 |
| Salivation (3) | 56 (66.7) | 1.23 |
| Swallowing (4) | 40 (47.6) | 0.81 |
| Feeding (5) | 39 (46.4) | 0.93 |
| Dressing (6) | 27 (32.1) | 0.64 |
| Taking a bath or shower (7) | 37 (44.0) | 0.77 |
| Grooming (8) | 39 (46.4) | 0.68 |
| Toilet use (9) | 34 (40.5) | 0.62 |
| Speech (10) | 59 (70.2) | 1.36 |
| Facial expression – oromandibular dystonia (11A) | 34 (40.5) | 0.69 |
| Facial expression – hypomimia (11B) | 40 (47.6) | 0.75 |
| Tremor at rest (12) |  |  |
| Left arm | 14 (16.7) | 0.24 |
| Right arm | 15 (17.9) | 0.27 |
| Left leg | 8 (9.5) | 0.15 |
| Right leg | 8 (9.5) | 0.20 |
| Head tremor (13) | 8 (9.5) | 0.15 |
| Rigidity (14) |  |  |
| Neck | 40 (47.6) | 0.89 |
| Left arm | 26 (31.0) | 0.71 |
| Right arm | 27 (32.1) | 0.70 |
| Left leg | 40 (47.6) | 1.11 |
| Right leg | 36 (42.9) | 1.00 |
| Finger taps (15) |  |  |
| Left | 63 (75.0) | 1.38 |
| Right | 63 (75.0) | 1.38 |
| Rapid alternating movements of hands (16) |  |  |
| Left | 68 (81.0) | 1.45 |
| Right | 64 (76.2) | 1.38 |
| Handwriting (17) | 52 (61.9) | 1.23 |
| Postural tremor in arms (18A) |  |  |
| Left | 35 (41.7) | 0.71 |
| Right | 42 (50.0) | 0.82 |
| Wing-beating tremor (18B) |  |  |
| Left | 5 (6.0) | 0.13 |
| Right | 5 (6.0) | 0.14 |
| Finger-to-nose test (19) |  |  |
| Left | 41 (48.8) | 0.77 |
| Right | 34 (40.5) | 0.76 |
| Leg agility (20) |  |  |
| Left | 52 (61.9) | 1.13 |
| Right | 51 (60.7) | 1.05 |
| Postural tremor in legs (21) |  |  |
| Left | 21 (25.0) | 0.43 |
| Right | 22 (26.2) | 0.51 |
| Cervical dystonia (22) | 39 (46.4) | 0.70 |
| Arm and hand dystonia (23) |  |  |
| Left | 47 (56.0) | 1.06 |
| Right | 46 (54.8) | 1.06 |
| Arising from chair (24) | 22 (26.2) | 0.65 |
| Posture – trunk dystonia (25A) | 26 (31.0) | 0.46 |
| Posture – ataxia of stance (25B) | 25 (29.8) | 0.52 |
| Posture – parkinsonism (25C) | 12 (14.3) | 0.24 |
| Gait – leg dystonia (26A) | 50 (59.5) | 1.12 |
| Gait – ataxia (26B) | 49 (58.3) | 1.19 |
| Gait – parkinsonism (26C) | 27 (32.1) | 0.54 |
| Chorea (27) |  |  |
| Face | 4 (4.8) | 0.12 |
| Trunk | 2 (2.4) | 0.05 |
| Left arm | 10 (11.9) | 0.19 |
| Right arm | 8 (9.5) | 0.17 |
| Left leg | 9 (10.7) | 0.17 |
| Right leg | 8 (9.5) | 0.18 |
