# Supplementary material for: Assessment of neurological symptoms and associated factors in patients with Wilson’s disease in Southwest China
Source: Orphanet J Rare Dis. 2025 Jul 4;20:342. doi: 10.1186/s13023-025-03874-2 (PMC12228280; doi:10.1186/s13023-025-03874-2)
Supplement: Supplementary file 4 — Additional file4 [file 13023_2025_3874_MOESM4_ESM.docx]

**Supplementary Table 4** UWDRS Part I scores based on characteristics of patients with WD.

| **Characteristics** | **Score [*M* (*Q*_1_, *Q*_3_)]** | ***p*** |
| --- | --- | --- |
| **Demographic Characteristics** |  |  |
| Sex |  |  |
| Male | 19.0 (4.3, 40.5) | 0.712 |
| Female | 20.0 (1.5, 51.5) |  |
| Age (years) |  |  |
| ≤20 | 47.0 (0.0, 73.0) | 0.390 |
| 21-30 | 14.5 (0.0, 36.0) |  |
| 31-40 | 29.5 (6.3, 50.0) |  |
| >40 | 15.0 (5.0, 26.5) |  |
| Registered residence type |  |  |
| Urban | 13.0 (0.0, 29.5) | **0.013** |
| Rural | 27.0 (6.3, 51.8) |  |
| BMI (kg/m^2^) |  |  |
| <18.5 | 35.0 (11.5, 53.0) | **0.009** |
| 18.5-24 | 16.0 (3.3, 40.5) |  |
| ≥24 | 7.0 (0.0, 20.0) |  |
| Education level |  |  |
| Junior high school and below | 29.5 (6.5, 51.3) | **0.001** |
| Senior high school | 34.5 (10.8, 69.0) |  |
| College and above | 6.0 (0.0, 21.0) |  |
| Marital status |  |  |
| Unmarried | 15.5 (0.0, 52.3) | 0.284 |
| Married | 17.0 (4.0, 36.5) |  |
| Divorced or widowed | 30.5 (20.3, 42.8) |  |
| Occupation |  |  |
| Personnel of service industries | 14.0 (1.0, 19.0) | **<0.001** |
| Personnel of enterprises or institutions | 5.0 (0.0, 16.8) |  |
| Student | 0.0 (0.0, 55.3) |  |
| Others | 12.5 (3.5, 18.8) |  |
| Unemployed | 35.5 (17.5, 61.5) |  |
| Family per capita monthly income (CNY) |  |  |
| ≤2500 | 33.0 (13.0, 60.0) | **<0.001** |
| 2500-5000 | 20.0 (5.0, 47.0) |  |
| >5000 | 4.0 (0.0, 16.0) |  |
| **Lifestyle Habits** |  |  |
| Physical exercise (times/week) |  |  |
| <1 | 25.0 (6.0, 51.0) | 0.058 |
| 1-4 | 3.5 (0.0, 46.5) |  |
| ≥5 | 15.0 (8.3, 34.8) |  |
| Sleep duration (hours) |  |  |
| <6 | 26.0 (11.8, 53.0) | 0.130 |
| 6-7 | 20.0 (2.5, 50.5) |  |
| ≥8 | 12.5 (0.0, 34.5) |  |
| Sleep quality |  |  |
| Good | 15.5 (0.5, 45.8) | 0.050 |
| Moderate | 6.5 (0.0, 35.3) |  |
| Poor | 30.0 (15.0, 54.0) |  |
| Smoking status |  |  |
| Yes | 12.5 (3.8, 34.5) | 0.456 |
| No | 19.0 (2.0, 50.0) |  |
| Drinking status |  |  |
| Yes | 17.0 (5.8, 36.0) | 0.709 |
| No | 19.0 (1.0, 50.0) |  |
| **Disease-related Information** |  |  |
| Positive family history |  |  |
| Yes | 17.0 (3.3, 43.3) | 0.794 |
| No | 19.0 (0.0, 46.5) |  |
| Age at onset (years) |  |  |
| ≤15 | 20.0 (0.0, 51.0) | 0.495 |
| 16-20 | 25.0 (5.5, 53.8) |  |
| 21-25 | 13.0 (0.5, 28.8) |  |
| >25 | 15.0 (4.0, 37.5) |  |
| Initial clinical subtype |  |  |
| Neurologic | 29.0 (15.0, 52.0) | **<0.001** |
| Non-neurologic | 0.0 (0.0, 22.5) |  |
| Onset to diagnosis (years) |  |  |
| <1 | 17.0 (0.0, 36.0) | 0.328 |
| 1-2 | 22.0 (4.5, 64.0) |  |
| >2 | 20.0 (5.0, 51.5) |  |
| Misdiagnosed |  |  |
| Yes | 15.0 (0.0, 45.0) | 0.202 |
| No | 24.5 (3.8, 47.8) |  |
| Disease duration (years) |  |  |
| ≤5 | 7.0 (0.0, 48.5) | 0.595 |
| 6-10 | 20.0 (1.5, 51.0) |  |
| 11-15 | 19.0 (7.0, 42.0) |  |
| 16-20 | 19.0 (4.8, 41.3) |  |
| >20 | 34.0 (13.0, 49.0) |  |
| Years of treatment (years) |  |  |
| ≤5 | 12.0 (0.0, 50.0) | 0.372 |
| 6-10 | 18.0 (0.0, 44.3) |  |
| 11-15 | 30.0 (13.0, 55.5) |  |
| 16-20 | 11.5 (3.0, 40.3) |  |
| >20 | 31.0 (13.0, 43.5) |  |
| **Treatment and Adherence** |  |  |
| Intravenous chelating agent |  |  |
| DMPS | 19.0 (4.0, 47.0) | 0.313 |
| EDTA | 14.0 (0.0, 34.0) |  |
| Oral chelating agent |  |  |
| DPA | 27.0 (6.8, 36.5) | 0.624 |
| DMSA | 16.0 (0.0, 42.3) |  |
| Zn | 29.5 (17.0, -) |  |
| DPA+DMSA | 49.0 (3.0, 103.3) |  |
| DPA+Zn | 15.0 (1.0, 34.0) |  |
| DPA+DMSA+Zn | 20.0 (9.5, 57.0) |  |
| DMSA+Zn | 19.0 (0.0, 59.5) |  |
| None | 5.0 (0.0, 21.8) |  |
| Regular medication |  |  |
| Completely | 20.0 (6.0, 51.0) | 0.402 |
| Mostly | 16.0 (0.0, 46.5) |  |
| Occasionally or not at all | 15.0 (0.0, 36.5) |  |
| Regular review |  |  |
| Completely | 15.5 (0.0, 46.3) | 0.307 |
| Mostly | 20.0 (7.0, 44.5) |  |
| Occasionally or not at all | 31.5 (6.5, 67.8) |  |
| Adherence to low-copper diets |  |  |
| Completely | 28.0 (8.5, 53.0) | **0.001** |
| Mostly | 6.0 (0.0, 26.3) |  |
| Occasionally or not at all | 45.0 (6.0, 100.0) |  |
| **Psychosocial Factors** |  |  |
| Social support |  |  |
| High | 7.0 (0.0, 18.0) | 0.062 |
| Medium | 20.0 (4.0, 51.0) |  |
| Low | 6.0 (4.0, -) |  |
| Mental health |  |  |
| Good | 13.0 (1.3, 28.0) | **0.004** |
| Moderate | 28.5 (0.0, 51.3) |  |
| Poor | 22.0 (2.0, 62.5) |  |
| Bad | 57.5 (32.5, 101.5) |  |
| **Liver Function** |  |  |
| Cirrhosis |  |  |
| Yes | 16.0 (1.0, 36.0) | 0.332 |
| No | 22.0 (3.5, 52.3) |  |
| Child-Pugh class |  |  |
| A | 20.0 (4.0, 47.0) | 0.274 |
| B | 6.5 (0.0, 17.5) |  |
| C | 19.0 (0.0, 65.0) |  |
| ALBI grade |  |  |
| 1 | 20.0 (4.5, 51.0) | **0.031** |
| 2 | 20.0 (0.0, 35.0) |  |
| 3 | 0.0 (0.0, 9.5) |  |
| **Copper Metabolism** |  |  |
| U-Cu (μg/L) |  |  |
| ≤100 | 17.0 (4.3, 37.3) | 0.939 |
| 100-200 | 27.5 (0.3, 45.8) |  |
| 200-500 | 13.0 (3.5, 34.0) |  |
| 500-1000 | 23.5 (0.0, 52.3) |  |
| >1000 | 19.5 (0.0, 53.8) |  |
| CP (g/L) |  |  |
| ≤0.02 | 20.0 (4.0, 60.0) | 0.660 |
| 0.02-0.06 | 16.0 (0.8, 34.3) |  |
| 0.06-0.1 | 19.5 (3.3, 48.0) |  |
| >0.1 | 28.0 (5.5, 42.3) |  |

All the examined continuous variables failed to conform to the normal distribution, thus results were presented as median (interquartile range).

BMI, body mass index; CNY, Chinese Yuan; DMPS, sodium dimercaptopropanesulfonate; EDTA, calcium sodium edetate; DPA, D-penicillamine; DMSA, dimercaptosuccinic acid; Zn, zinc; ALBI, albumin-bilirubin; U-Cu, urinary copper; CP, ceruloplasmin.

Bold: results significant (*p*＜0.05).
