# Supplementary material for: Assessment of neurological symptoms and associated factors in patients with Wilson’s disease in Southwest China
Source: Orphanet J Rare Dis. 2025 Jul 4;20:342. doi: 10.1186/s13023-025-03874-2 (PMC12228280; doi:10.1186/s13023-025-03874-2)
Supplement: Supplementary file 5 — Additional file5 [file 13023_2025_3874_MOESM5_ESM.docx]

**Supplementary Table 5** The intermediate iteration process in stepwise multiple linear regression.

| **The number of iterations** | **Variable** | ***β*** | ***SE*** | ***t*** | ***p*** |
| --- | --- | --- | --- | --- | --- |
| 1 | Constant | 37.571 | 3.684 | 10.198 | <0.001 |
|  | Adherence to low-copper diets (mostly) | -22.137 | 5.671 | -3.903 | <0.001 |
| 2 | Constant | 42.970 | 3.836 | 11.202 | <0.001 |
|  | Adherence to low-copper diets (mostly) | -19.707 | 5.444 | -3.620 | <0.001 |
|  | Family per capita monthly income (>5000) | -20.007 | 5.758 | -3.474 | 0.001 |
| 3 | Constant | 40.014 | 3.837 | 10.428 | <0.001 |
|  | Adherence to low-copper diets (mostly) | -17.898 | 5.292 | -3.382 | 0.001 |
|  | Family per capita monthly income (>5000) | -18.941 | 5.572 | -3.400 | 0.001 |
|  | Mental health (bad) | 33.626 | 11.401 | 2.949 | 0.004 |
| 4 | Constant | 42.207 | 3.830 | 11.021 | <0.001 |
|  | Adherence to low-copper diets (mostly) | -18.696 | 5.161 | -3.622 | <0.001 |
|  | Family per capita monthly income (>5000) | -14.694 | 5.665 | -2.594 | 0.011 |
|  | Mental health (bad) | 33.791 | 11.100 | 3.044 | 0.003 |
|  | Occupation (personnel of enterprises or institutions) | -17.602 | 6.764 | -2.602 | 0.011 |
| 5 | Constant | 45.396 | 3.902 | 11.634 | <0.001 |
|  | Adherence to low-copper diets (mostly) | -18.438 | 5.013 | -3.678 | <0.001 |
|  | Family per capita monthly income (>5000) | -15.184 | 5.504 | -2.759 | 0.007 |
|  | Mental health (bad) | 31.119 | 10.824 | 2.875 | 0.005 |
|  | Occupation (personnel of enterprises or institutions) | -20.467 | 6.654 | -3.076 | 0.003 |
|  | Occupation (personnel of service industries) | -20.690 | 7.664 | -2.699 | 0.008 |
| 6 | Constant | 48.480 | 4.053 | 11.962 | <0.001 |
|  | Adherence to low-copper diets (mostly) | -17.012 | 4.951 | -3.436 | 0.001 |
|  | Family per capita monthly income (>5000) | -12.199 | 5.548 | -2.199 | 0.030 |
|  | Mental health (bad) | 29.274 | 10.637 | 2.752 | 0.007 |
|  | Occupation (personnel of enterprises or institutions) | -20.508 | 6.520 | -3.145 | 0.002 |
|  | Occupation (personnel of service industries) | -20.581 | 7.511 | -2.740 | 0.007 |
|  | Initial clinical subtype (non-neurologic) | -11.802 | 5.142 | -2.295 | 0.024 |
| 7 | Constant | 50.118 | 4.060 | 12.345 | <0.001 |
|  | Adherence to low-copper diets (mostly) | -14.673 | 4.993 | -2.939 | 0.004 |
|  | Family per capita monthly income (>5000) | -11.107 | 5.480 | -2.027 | 0.045 |
|  | Mental health (bad) | 27.621 | 10.490 | 2.633 | 0.010 |
|  | Occupation (personnel of enterprises or institutions) | -23.496 | 6.566 | -3.579 | 0.001 |
|  | Occupation (personnel of service industries) | -23.415 | 7.506 | -3.119 | 0.002 |
|  | Initial clinical subtype (non-neurologic) | -12.154 | 5.059 | -2.402 | 0.018 |
|  | Occupation (others) | -16.921 | 7.997 | -2.116 | 0.037 |

Variables enter the model when *p* < 0.05 and are excluded when *p* > 0.1.
